# MINOCA (Myocardial Infarction With Non-obstructive Coronary Arteries) Due to Diffuse Coronary Microvascular Dysfunction in a Patient With Systemic Autoimmune Disease and Chronic Kidney Disease

**DOI:** 10.7759/cureus.103816

**Published:** 2026-02-18

**Authors:** Teenu Kamboj, Shubhkaran Singh Gill, Neha Chopra, Gurpreet S Wander

**Affiliations:** 1 Department of Internal Medicine, Dayanand Medical College and Hospital, Ludhiana, IND; 2 Department of General Surgery, Dayanand Medical College and Hospital, Ludhiana, IND; 3 Department of Cardiology, Safdarjung Hospital, New Delhi, IND; 4 Department of Cardiology, Dayanand Medical College and Hospital, Ludhiana, IND

**Keywords:** acetylcholine provocation testing, antineutrophil cytoplasmic antibody (anca) associated vasculitis (aav), autoimmune disease, coronary flow reserve, coronary microvascular dysfunction (cmd), endothelial dysfunction, microvascular angina, myocardial infarction with non-obstructive coronary arteries (minoca), positron emission tomography computed tomography, st-elevation myocardial infarction (stemi) mimic

## Abstract

Myocardial infarction with non-obstructive coronary arteries (MINOCA) represents a heterogeneous entity requiring systematic exclusion of alternative diagnoses and identification of the underlying mechanism. Coronary microvascular dysfunction (CMD) is an important but often underdiagnosed cause. Systemic inflammatory flares may precipitate microvascular ischemia, yet direct multimodality confirmation remains limited. A 51-year-old woman with active perinuclear anti-neutrophil cytoplasmic antibody (P-ANCA) vasculitis, rheumatoid arthritis, and stage 3b chronic kidney disease presented with acute chest pain, dynamic ST-segment changes, and a rise in high-sensitivity troponin T (peak 2,526 pg/mL), fulfilling criteria for acute myocardial infarction. Coronary angiography demonstrated non-obstructive coronary arteries (<50% stenosis). Cardiac magnetic resonance imaging (including T1 mapping, T2 mapping, and LGE) excluded myocarditis, stress cardiomyopathy, and infiltrative disease. Invasive coronary physiology revealed impaired coronary flow reserve (CFR 1.7) and an elevated index of microvascular resistance (IMR 32). Acetylcholine provocation excluded epicardial vasospasm. ^13^N-ammonia positron emission tomography (PET) confirmed reduced global stress myocardial blood flow and global myocardial flow reserve (1.8), consistent with diffuse CMD. The inflammatory surge (leukocytosis, raised ESR, raised CRP) temporally paralleled troponin elevation. A diagnosis of inflammatory-triggered MINOCA due to diffuse CMD was established. Immunosuppressive therapy was optimized alongside mechanism-directed antianginal therapy. Dual antiplatelet therapy was limited to a short course given the absence of plaque rupture and elevated bleeding risk. At the six-month follow-up, inflammatory markers and troponin normalized, and the patient remained symptom-free. This case demonstrates an inflammatory endotype of MINOCA, where acute autoimmune activation precipitated diffuse CMD. Multimodality confirmation with invasive physiology and PET imaging established the mechanism beyond exclusion. Mechanism-directed therapy targeting the inflammatory substrate, in addition to tailored cardiovascular management, resulted in clinical stabilization.

## Introduction

Myocardial infarction with non-obstructive coronary arteries (MINOCA) is defined as an acute myocardial infarction in the absence of obstructive coronary artery disease (i.e., no epicardial stenosis ≥50%), and alternative causes of myocardial injury, such as myocarditis, stress cardiomyopathy, pulmonary embolism, or other non-ischemic etiologies, are systematically excluded [1]. MINOCA accounts for approximately 5% to 10% of all acute myocardial infarctions [1]. The diagnosis of MINOCA requires a shift from anatomical to functional assessment. Systemic autoimmune diseases and chronic kidney disease (CKD) can lower the ischemic threshold through endothelial injury, chronic inflammation, and oxidative stress. Chronic systemic inflammation impairs endothelial nitric oxide production and promotes oxidative stress within the coronary microcirculation. This results in increased microvascular tone and reduced vasodilatory capacity, predisposing to ischemia even in the absence of epicardial obstruction [2,3].

We report a patient with overlapping systemic autoimmune disease and stage 3b CKD who developed MINOCA with objective evidence of diffuse coronary microvascular dysfunction (CMD). This case uniquely demonstrates CMD as the primary ischemic mechanism using both invasive coronary functional testing and quantitative ¹³N-ammonia positron emission tomography (¹³N-PET) myocardial perfusion imaging, underscoring how inflammatory and renal comorbidities act as microvascular multipliers despite angiographically normal epicardial coronary arteries.

## Case presentation

A 51-year-old postmenopausal woman presented with acute, non-pleuritic, non-positional substernal chest pain at rest beginning two hours prior to admission, associated with nausea and vomiting. On presentation, her vital signs were stable with a blood pressure of 128/78 mmHg, a heart rate of 84 bpm, and an oxygen saturation of 98% at room temperature. She was afebrile. Systemic examination was within normal limits with no signs of heart failure, active vasculitis, or volume overload. She had no prior history of obstructive coronary disease, atrial fibrillation, or thromboembolism. Her medical history included perinuclear anti-neutrophil cytoplasmic antibody (P-ANCA) vasculitis, rheumatoid arthritis, stage 3b CKD, and long-standing hypertension. Prior to her admission, the patient was already being managed with a triple-drug immunosuppressive regimen for her underlying P-ANCA vasculitis and rheumatoid arthritis. This home regimen consisted of mycophenolate mofetil (Cellcept) 500 mg taken twice daily, leflunomide (Letra) 20 mg once daily, and low-dose prednisolone (Wysolone) at 5 mg once daily.

**Figure 1:**
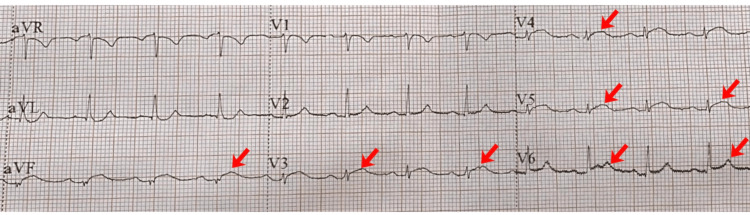
Electrocardiogram Electrocardiogram showing sinus rhythm with transient mild ST-segment elevation in inferior leads and leads V3-V6 (as indicated by arrows).

An electrocardiogram showed sinus rhythm with transient mild ST-segment elevation in the inferior leads and leads V3-V6 (Figure 1).

High-sensitivity cardiac troponin T peaked at 2,526 pg/mL, correlating with a systemic inflammatory surge (Table 1). Laboratory testing showed mild normocytic anemia (Hb 10.20 g/dL (reference range: 12.0-15.5 g/dL); MCV 80.50 fL (reference range: 80-100 fL); ESR 46.5 mm/hr (reference range: 0-20 mm/hr)) contributing to reduced oxygen carrying capacity and renal indices showing a transient renal fluctuation (creatinine 1.54 mg/dL (reference range: 0.6-1.1 mg/dL); eGFR 41 mL/min/1.73 m² (reference range: >59 mL/min/1.73 m²)).

**Table 1:** Measurements of Troponin T, Total Leukocyte Count, eGF, and CRP This table shows the changes in troponin T levels (pg/mL), total leukocyte count (×10³/µL), and eGFR at different timepoints from admission. Troponin T peaked at 12 hours, while total leukocyte count and eGFR trends over the same period. C-reactive protein is highly elevated during the acute phase, suggesting a systemic inflammatory response. The dashes indicate values were not measured.

Timepoint	Troponin T (pg/mL)	Total Leukocyte Count (×10³/µL)	eGFR (mL/min/1.73 m²)	C-Reactive Protein (mg/L)
Reference Range	<10	4.0–11.0	>59	<1.0
Admission (0 h)	57.3	12.99	41	-
6 h	1,118.0	-	-	-
12 h (Peak)	2,526.0	23.00	39.3	54.5

**Figure 2:**
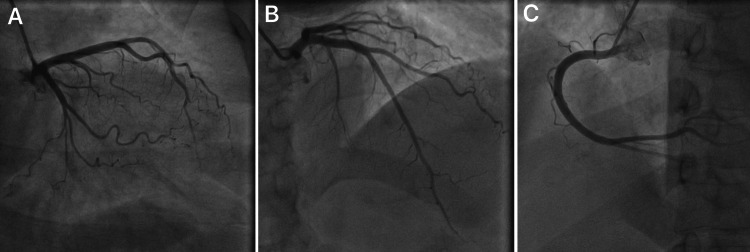
Coronary Angiography A: The left main (LM) coronary artery bifurcating into the left anterior descending (LAD) and left circumflex (LCx) arteries. The vessels appear widely patent with smooth luminal contours and no evidence of obstructive stenosis or filling defects. B: A longitudinal view of the LAD and its diagonal branches. The vessel maintains a consistent caliber throughout its mid-to-distal segments, showing no focal narrowing or atherosclerotic plaque. C: The right coronary artery (RCA) in its entirety, exhibiting a typical "C-loop" morphology. The vessel is non-obstructive with preserved flow to the distal branches.

Transthoracic echocardiography with Doppler evaluation showed normal left ventricular (LV) size and function (ejection fraction 60%), no regional wall-motion abnormalities, normal diastolic filling parameters, and no significant valvular disease. Coronary angiography, performed for suspected inferior STEMI (ST-segment elevation myocardial infarction), demonstrated non-obstructive coronary arteries with mild diffuse atherosclerotic changes and no stenosis ≥ 50%, no dissection or thrombus (Figures 2A-C). LV angiography showed preserved systolic function.

**Figure 3:**
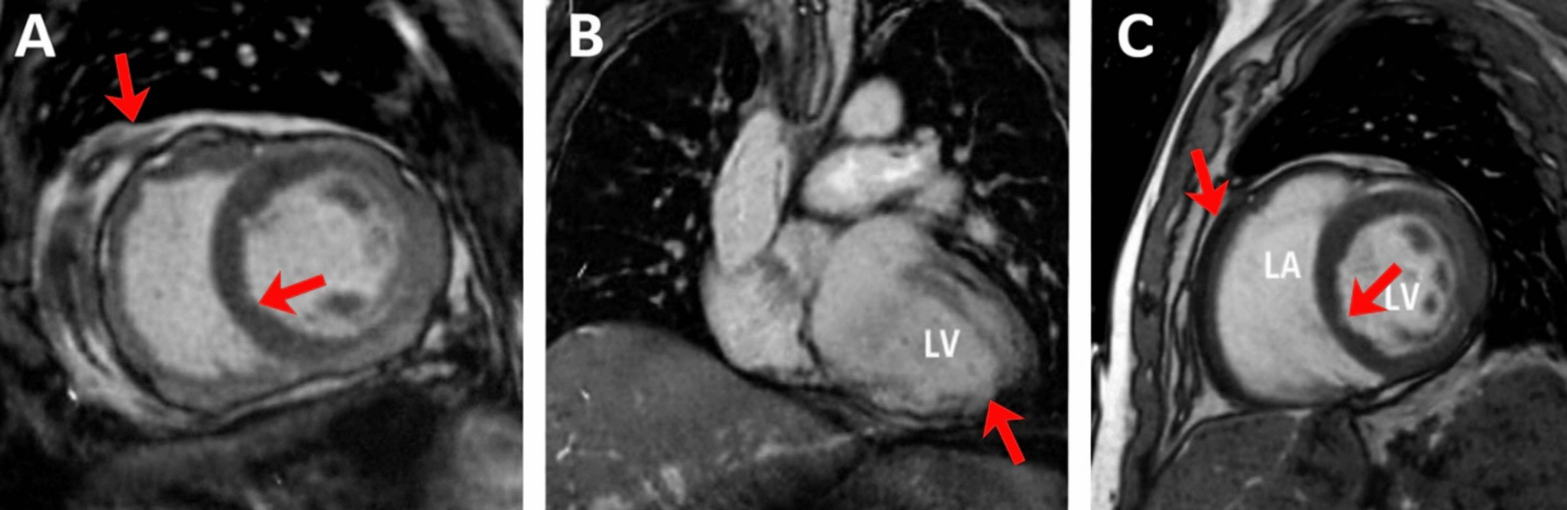
Cardiac Magnetic Resonance Imaging A: Short-axis view: a cross-sectional slice of the left ventricle (LV) and right ventricle (RV) at the mid-ventricular level (as indicated by arrows). The myocardial walls show uniform thickness without evidence of gross structural abnormalities. B: Coronal view: a longitudinal orientation showing the LV and the aortic outflow tract. The image displays normal cardiac positioning and the relationship between the major chambers and the ascending aorta (as indicated by arrows). C: Labeled short-axis view: a repeat of the short-axis perspective with the LV and the RV (labeled as 'LA' on the right side) cavities identified (as indicated by arrows). The myocardium appears dark (isointense), indicating a lack of focal hyperintensities or late gadolinium enhancement.

Cardiac magnetic resonance (CMR) imaging showed no myocardial edema, late gadolinium enhancement, stress cardiomyopathy, or infiltrative disease (Figures 3A-C).

**Figure 4:**
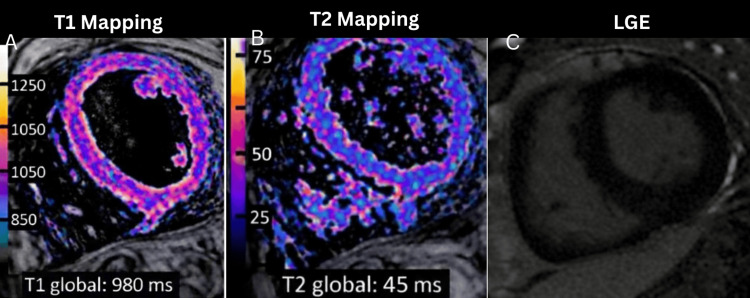
Cardiac MRI Tissue Characterization (T1, T2, and LGE Mapping) A: The native T1 parametric map demonstrates a global T1 value of 980 ms. At a field strength of 1.5T, the typical reference range for healthy myocardium is approximately 950–1050 ms. The myocardium exhibits a homogeneous signal without evidence of significant T1 prolongation. Under the Lake Louise Criteria, a normal native T1 value suggests the absence of diffuse interstitial expansion, which would typically be seen in the presence of myocardial edema or fibrosis. The pixel-wise map shows no focal areas of increased T1, consistent with normal myocardial tissue composition. B: Quantitative T2 mapping reveals a global T2 value of 45 ms. The established reference range for normal myocardium at 1.5T is generally 40–50 ms. There is no evidence of myocardial edema, as the T2 values remain well below the pathological threshold (typically >52-55 ms at 1.5T). The color-coded map confirms a uniform distribution of T2 relaxation times across the short-axis slice, with no focal "hot spots" in the subepicardial or mid-wall layers. This represents a negative T2-based criterion for active inflammatory edema. C: Post-contrast T1-weighted inversion recovery imaging shows no evidence of late gadolinium enhancement. There are no visible areas of focal hyperenhancement in the subepicardial or intramural distributions, which are the hallmark signatures of myocardial necrosis or replacement fibrosis in myocarditis. This finding satisfies the negative T1-based criterion for focal myocardial injury.

At 1.5T, the global T2 (45 ms) and native T1 (980 ms) values fall within the normal reference ranges of 40-50 ms and 950-1050 ms, respectively. The absence of T1/T2 prolongation and focal late gadolinium enhancement (LGE) results in a negative study for acute myocarditis per the 2018 Lake Louise Criteria (Figure 4). Complementing these findings, the extracellular volume (ECV) was calculated at 26.4% (hematocrit = 40.1%). In healthy myocardium, the ECV typically ranges between 23% and 28%. An ECV of 26.4% indicates a normal cellular-to-interstitial ratio, providing strong evidence against diffuse pathologies such as interstitial fibrosis, systemic amyloid deposition, or significant myocardial edema.

Invasive coronary functional testing was performed during the index hospitalization following stabilization with minimized contrast volume and renal protective measures. The coronary flow reserve measured in the left anterior descending artery during adenosine-induced hyperemia was reduced at 1.7 (normal >2.0), and the index of microvascular resistance was elevated at 32 units (normal <25). Invasive testing was performed despite CKD for diagnostic necessity and mechanism clarification. Intracoronary acetylcholine was administered in incremental doses over 30 seconds up to 100 micrograms in the left coronary artery and 80 micrograms in the right coronary artery. No ≥90% epicardial vasoconstriction was observed angiographically. However, chest pain with transient ischemic ECG changes occurred. These findings indicate endothelium-dependent microvascular dysfunction as the mechanism of ischemia, in the absence of epicardial coronary spasm.

**Figure 5:**
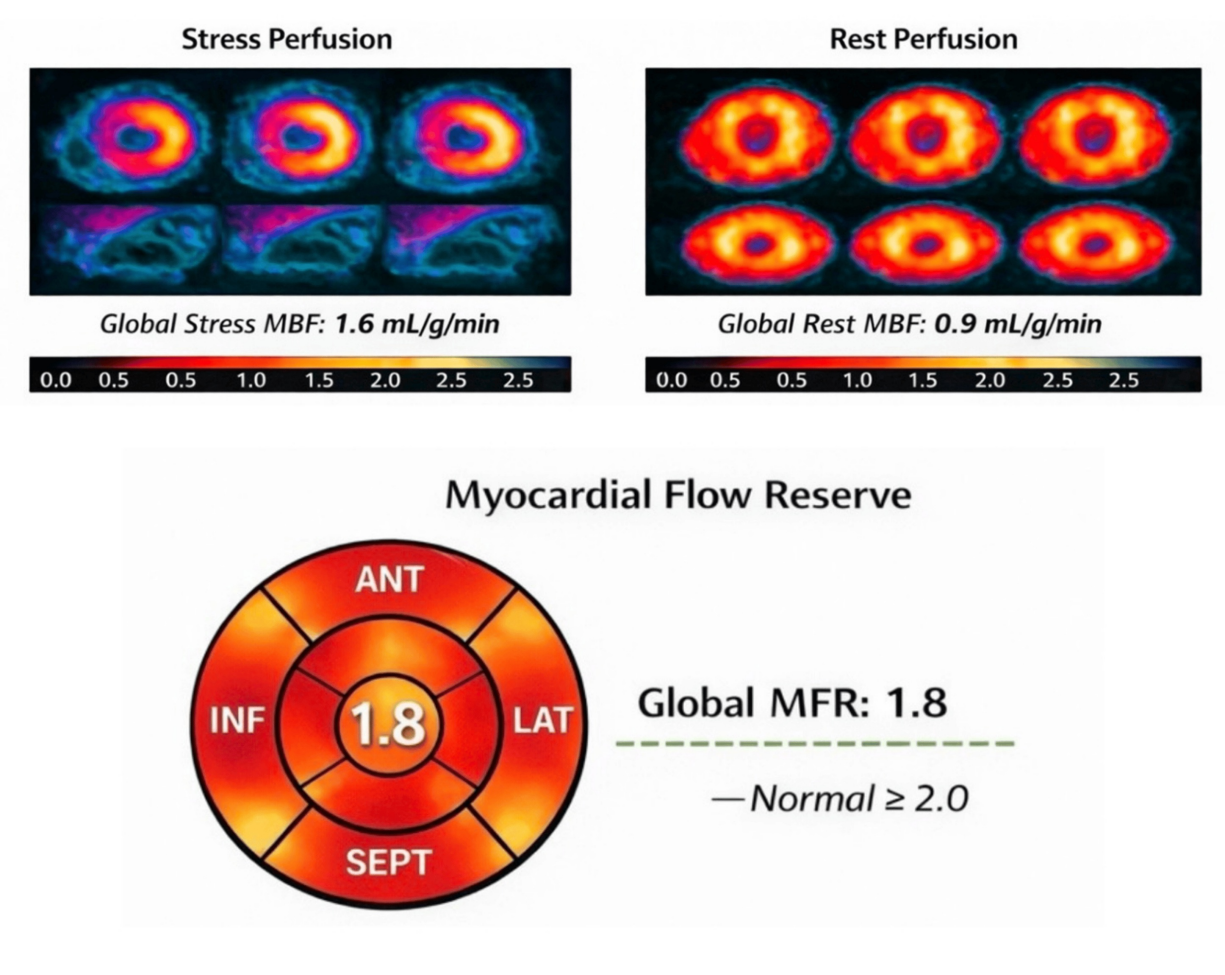
¹³N-Ammonia Positron Emission Tomography Myocardial Perfusion Imaging Quantitative stress/rest perfusion PET demonstrating globally reduced stress myocardial blood flow (1.6 mL/g/min) with preserved rest perfusion (0.9 mL/g/min) and reduced myocardial flow reserve (1.8), consistent with diffuse coronary microvascular dysfunction. Image created by the authors.

¹³N-PET myocardial perfusion imaging was performed on day 2 of hospitalization and demonstrated reduced global stress myocardial blood flow (1.6 mL/g/min) and a global myocardial flow reserve of 1.8 (normal ≥ 2.0), without regional perfusion defects, which are consistent with diffuse CMD (Figure 5).

MINOCA due to microvascular angina was diagnosed. Normal CMR excluded myocarditis, preserved ventricular function excluded Takotsubo, and acetylcholine testing showed no ≥90% epicardial spasm, making vasospasm unlikely. These findings were consistent with diffuse CMD as the predominant mechanism.

The patient was managed with a mechanism-oriented, individualized approach in consultation with rheumatology. Her baseline immunosuppressive regimen of mycophenolate mofetil (500 mg BD), leflunomide (20 mg), and low-dose prednisolone (5 mg) was maintained to address the systemic flare. Hemodynamic therapy with beta-blockers and calcium channel blockers (metoprolol 50 mg/amlodipine 5 mg, with supplemental amlodipine 2.5 mg) was titrated to improve diastolic filling and reduce the supply-demand mismatch from tachycardia and anemia. Given the absence of obstructive disease and CKD-related bleeding risk, dual antiplatelet therapy (aspirin 150 mg + clopidogrel 75 mg) was limited to a short course. Despite a baseline LDL of 70 mg/dL and the absence of obstructive CAD, atorvastatin (40 mg HS) was initiated for secondary prevention and endothelial stabilization. Isosorbide dinitrate (5 mg S/L) was utilized for breakthrough angina to counteract transient microvascular spasm. As a nitric oxide donor, it promotes vascular smooth muscle relaxation and reduces microvascular tone, thereby improving myocardial perfusion and relieving ischemic symptoms.

Outcome and follow-up

At the three-month follow-up, the patient remained free from recurrent chest pain or heart failure symptoms. High-sensitivity troponin levels had normalized, alongside a complete normalization of inflammatory markers (ESR and CRP). Repeat invasive coronary physiology at three months demonstrated a CFR of 2.5 and an IMR of 18. While these values suggest a significant reversibility of microvascular dysfunction, it is important to note that IMR and CFR are hemodynamically influenced parameters; their normalization likely reflects the direct pharmacological effect of the patient’s maintained regimen of amlodipine and isosorbide dinitrate, which effectively modulated microvascular tone and reduced spasm. This functional improvement paralleled the resolution of the systemic inflammatory flare and symptom relief. Invasive assessment enabled the direct measurement of these indices, which would not have been possible with PET imaging, while contrast volume was minimized and renal protective measures were strictly employed. The patient's clinical status remained stable at six months, with no further acute coronary events, confirming the efficacy of a mechanism-oriented approach in managing microvascular ischemia in the setting of systemic inflammation.

## Discussion

Defining the inflammatory endotype of MINOCA

This case illustrates the transition of MINOCA from a diagnosis of exclusion to a mechanism-defined entity. Contemporary scientific statements emphasize that MINOCA requires identification of a specific underlying substrate rather than remaining a working diagnosis [1,4,5]. In this patient, the clinical presentation of an inferior STEMI-mimic occurred in the context of long-standing P-ANCA vasculitis and rheumatoid arthritis. Systemic inflammatory disorders are increasingly recognized as contributors to myocardial injury and MINOCA through immune-mediated vascular dysfunction [6,7]. Unlike type-1 myocardial infarction, which involves plaque rupture and thrombosis, the inflammatory endotype is characterized by diffuse CMD, particularly in women and in patients with autoimmune or renal comorbidities [7]. Large cohort and registry studies demonstrate that patients with systemic inflammatory diseases have significantly higher risks of MINOCA and INOCA, likely due to a chronically primed vascular bed with heightened endothelial sensitivity to cytokines and oxidative stress [6,7].

Molecular pathophysiology: the inflammasome and endothelial failure

The defining feature of the inflammatory endotype is the interaction between systemic cytokine activation and the coronary microvascular bed. Cytokines, including interleukin-1β, interleukin-6, and tumor necrosis factor-alpha, disrupt the endothelial glycocalyx, impair mechanotransduction, and reduce nitric oxide bioavailability through oxidative stress pathways [8,9]. Inflammatory signaling promotes endothelial nitric oxide synthase uncoupling, shifting the enzyme from nitric oxide production toward superoxide generation, with subsequent peroxynitrite formation and tetrahydrobiopterin depletion [10]. These mechanisms are central to endothelial dysfunction in inflammatory cardiovascular disease [8,10].

Acetylcholine-induced microvascular spasm confirmed a hyper-reactive microvascular state, consistent with CMD [11]. Neutrophil extracellular traps contribute to microvascular obstruction, no-reflow phenomena, and oxidative endothelial injury during inflammatory activation [12,13]. NETosis has been implicated as a mechanistic bridge between innate immune activation and CMD [12,13].

Structural remodeling and mechanical resistance

Beyond functional impairment, chronic inflammation promotes structural remodeling of the coronary microvasculature. Persistent TNF-alpha and IL-6 signaling induces vascular smooth muscle proliferation and perivascular fibrosis, increasing the wall-to-lumen ratio of resistance arterioles [8,14].

Elevated IMR reflects increased microvascular resistance, which may result from structural remodeling [15]. Studies demonstrate that elevated IMR is associated with impaired myocardial perfusion and adverse cardiovascular outcomes [15,16].

In CKD, capillary rarefaction, endothelial apoptosis, and fibrosis further impair coronary microvascular reserve and increase ischemic risk [17,18]. These structural and inflammatory alterations provide a mechanistic explanation for ischemia without obstructive epicardial disease [5,14].

Diagnostic synergy: integrating IMR and PET perfusion

IMR provides a reproducible invasive measure of microvascular resistance, while PET-derived myocardial flow reserve (MFR) reflects global hyperemic capacity [15,19]. An MFR below 2.0 independently predicts adverse cardiovascular outcomes and reflects impaired coronary vasodilatory reserve [19,20]. The combination of elevated IMR and reduced MFR confirms primary microvascular ischemia rather than isolated supply-demand mismatch.

Cardiac MRI may fail to detect diffuse microvascular dysfunction in the absence of focal fibrosis or edema, underscoring the complementary role of functional physiological testing in MINOCA [5]. Stratified diagnostic approaches improve symptom control and clinical outcomes in patients with CMD [21]. Limitations of this case include the absence of intravascular imaging (OCT/IVUS), although no epicardial disease was observed angiographically, and the fact that this is a single-patient case report, which may limit generalizability.

Reversibility and the targeted anti-inflammatory axis

Optimization of immunosuppressive therapy resulted in normalization of IMR and CFR. The normalization of CFR and IMR at three months likely reflects the reversal of functional microvascular dysregulation rather than fixed structural remodeling. Inflammatory activation and endothelial dysfunction associated with systemic autoimmune disease and CKD can impair nitric oxide bioavailability and increase microvascular tone, resulting in reduced hyperemic flow and elevated microvascular resistance. High-intensity statin therapy and optimization of vasomotor therapy may restore endothelial-dependent vasodilation, reduce oxidative stress, and attenuate inflammatory signaling, thereby improving coronary flow reserve and lowering the index of microvascular resistance [22]. The CANTOS trial demonstrated that IL-1β inhibition reduces recurrent cardiovascular events, establishing inflammation as a modifiable therapeutic target in atherosclerotic disease [9]. Statins further improve endothelial function through pleiotropic effects, including Rho-kinase inhibition and oxidative stress reduction [14,23].

Clinical implications for multidisciplinary management

Uniform acute coronary syndrome (ACS) protocols may be insufficient in patients with systemic inflammatory disorders. Mechanism-directed therapy guided by invasive physiology improves angina and quality of life, as demonstrated in the CorMicA trial [21]. Integrating pathophysiology, imaging, and invasive assessment allows the transformation of MINOCA into a mechanism-based entity requiring targeted treatment [4,5].

## Conclusions

MINOCA in the setting of systemic autoimmune disease and CKD represents a complex diagnostic and therapeutic challenge driven by CMD and endothelial injury. Comprehensive diagnostic evaluation, careful exclusion of alternative etiologies, and individualized multidisciplinary management are essential for optimal care in such patients.
